# Prevalence of metabolic syndrome in Murcia Region, a southern European Mediterranean area with low cardiovascular risk and high obesity

**DOI:** 10.1186/1471-2458-11-562

**Published:** 2011-07-14

**Authors:** Diana Gavrila, Diego Salmerón, José-Manuel Egea-Caparrós, José M Huerta, Alfonso Pérez-Martínez, Carmen Navarro, María-José Tormo

**Affiliations:** 1 Department of Epidemiology, Regional Authority for Health & Consumer Affairs (Consejería de Sanidad y Consumo), Ronda de Levante 11, 30008 Murcia, Spain; 2 CIBER Epidemiología y Salud Pública (CIBERESP), Spain; 3 Clinical Laboratory, Hospital Morales Meseguer, Avda. Marqués de los Vélez s/n, 30008 Murcia, Spain; 4 Department of Socio-health Sciences, University of Murcia School of Medicine, Spain

## Abstract

**Background:**

Metabolic syndrome (MS) is associated with subsequent appearance of diabetes and cardiovascular disease. As compared to other Spanish regions, Murcia (southern Spain) registers increased obesity as well as cardiovascular morbidity and mortality. The aim of this study was to assess the prevalence of MS and its components, awareness of obesity as a health risk and associated lifestyles.

**Methods:**

A population-based, cross-sectional study was conducted in 2003, covering a sample of 1555 individuals 20 years and over. MS was defined according to the Revised National Cholesterol Education Program Adult Treatment Panel III (R-ATPIII), International Diabetes Federation (IDF) and Joint Interim Statement (JIS) criteria. Both low (94/80) and high (102/88) waist circumference (WC) thresholds were considered.

**Results:**

Prevalence of MS was 27.2% (95%CI: 25.2-29.2), 32.2% (95%CI: 30.1-34.3) and 33.2% (95%CI: 31.2-35.3) according to the R-ATPIII, IDF and JIS94/80 respectively. It increased with age until reaching 52.6% (R-ATPIII) or 60.3% (JIS94/80) among persons aged 70 years and over, and was higher in persons with little or no formal education (51.7% R-ATPIII, 57.3% JIS94/80). The most common risk factors were hypertension (46.6%) and central obesity (40.7% and 66.1% according to high and low WC cut-off points respectively). Although most persons were aware that obesity increased health risks, regular exercise was very unusual (13.0% centrally obese, 27.2% non-centrally obese). Adherence to dietary recommendations was similar among centrally obese and non-centrally obese subjects.

**Conclusions:**

Prevalence of MS is high in our population, is comparable to that found in northern Europe and varies with the definition used. Adherence to preventive recommendations and to adequate weight promotion is very low. In the absence of a specific treatment for MS, integrated intervention based on a sustained increase in physical activity and changes in diet should be reinforced.

## Background

Metabolic syndrome (MS), a cluster of metabolically related cardiovascular risk factors, is associated with increased risk of death, cardiovascular events or diabetes [1]. Nowadays, estimates suggest that the population-attributable fraction for MS is approximately 6%-7% for all-cause mortality, 12%-17% for cardiovascular disease and 30%-52% for diabetes, depending on the criteria used [1]. Insulin resistance and central obesity seem to be closely involved in its aetiology [2,3].

Although the Murcia Region registers low cardiovascular mortality [4] and low incidence of acute myocardial infarction [5] at a European level, its myocardial infarction incidence and mortality rates are nevertheless among the highest in Spain [5,6]. Prevalence of diabetes in Murcia is considered to be at the upper limit of the moderate diabetes prevalence range [7], despite a notably high adherence to the Mediterranean dietary pattern and its components [8-10], something that has been shown to have a beneficial effect on insulin sensitivity [11]. Overweight and general obesity are very frequent and affect two out of every three adults [12]. Abdominal obesity is one of the highest recorded among European populations [13].

Bearing in mind the important role of central obesity in its appearance, a high prevalence of MS is expected in the adult population of the Murcia Region, thus justifying our interest in the matter. The main difficulty hindering its assessment is the lack of consensus on a definition. Currently, there are several definitions in more or less widespread use. There are: a) the World Health Organization definition [14] including a criterion of albuminuria, hardly available in large population-based surveys; b) the European Group for the Study of Insulin Resistance definition [15] focusing on insulin resistance with the expensive determination of insulinaemia; c) the revised National Cholesterol Education Program Adult Treatment Panel III (R-ATPIII) definition [16] and d) the International Diabetes Federation (IDF) definition [3], focusing on central obesity and using different cut-off points for waist circumference with the aim of being adaptable to different population. Recently, the major organizations involved in the clinical characterisation of MS (the IDF Task Force on Epidemiology and Prevention, the National Heart, Lung and Blood Institute, the American Heart Association, the World Heart Federation, the International Atherosclerosis Society and the International Association for the Study of Obesity) attempted to unify criteria by issuing a Joint Interim Statement (JIS)[17]. Accordingly, our study sought to assess the prevalence of the MS according to the R-ATPIII, IDF and JIS definitions, as well as the prevalence of each of its components, awareness of obesity as a health risk, and associated lifestyles.

## Methods

### Study Population

Murcia, a Mediterranean area lying in the south-east of Spain, is one of the 17 Autonomous Regions (*comunidades autónomas*) into which the country is divided. The framework for this study was the region's entire adult population aged 20 years and over, a total of 901,920 persons (2001 Census). Two thousand, five hundred and sixty-two subjects were selected by randomised sampling, stratified by age, sex, health area and type of residence (urban or rural), and invited to participate from July 2001 to June 2003. Exclusion criteria were pregnancy and/or any serious physical or mental disease. Subjects were invited to a primary health care centre, where they answered a lifestyle questionnaire administered by trained interviewers, underwent a physical examination, and provided a blood sample for biochemical determinations including fasting plasma glucose, HDL-cholesterol and triglycerides. Details on sampling, study design, biochemical determinations and physical examination have been published elsewhere [7]. The study was approved by the Ethics Committee of the Virgen de la Arrixaca University Teaching Hospital, the main regional hospital, and all subjects gave their written informed consent.

### Lifestyle questionnaire

To assess awareness of the pathogenic role of obesity, subjects were asked whether they considered it a disease, by answering the questions "In your opinion, is obesity a disease?" and "Do you know that obesity is associated with diabetes and heart attacks?" Questions on leisure-time physical activity were based on a previously validated questionnaire [18]. The number of hours of leisure-time physical activity per week was recorded. The American College of Sports Medicine and the American Heart Association recommend that adults should do physical activity for a minimum of 30 minutes a day, five days a week, in order to promote and maintain good health [19]. In line with these recommendations, moderate to vigorous physical activity for two and a half hours per week was considered adequate. Intake of the main dietary components (cereals, fruit, vegetables, legumes, fish, red meat, olive oil and wine) was measured using a food-frequency questionnaire. According to the Mediterranean diet pyramid [20], using olive oil daily, eating cereals, fruit, vegetables more than once daily, legumes and fish more than once per week and red meat less than twice per week was considered adequate. Insofar as alcohol was concerned, wine consumption of one glass per week to three per day for men and one glass per week to one per day for women was deemed to be the recommended moderate intake. Smoking was classified as never, ex- and current smoker. Educational level was defined as little or no formal education, primary, secondary and university.

### Physical examination

The participants underwent a physical examination, in which weight and height were measured using a stadiometer with an accuracy of 1 cm (Añó-Sayol model 1SB, Barcelona, Spain) and a digital scale with an accuracy of 100 g (TEFAL model SC-3301, GROUPE SEB Iberica, S.A., Barcelona, Spain). Body mass index (BMI) was calculated as weight divided by the square height, in kg/m2. Additionally, waist and hip circumferences were measured according to WHO recommendations [21]. Arterial blood pressure was measured according to the MONICA protocol [22], using a digital sphygmomanometer (BOSO oscillomat^®^). Systolic and diastolic arterial blood pressure was calculated as the mean of two consecutive measures.

### Definitions of the metabolic syndrome, impaired fasting glucose and diabetes

Participants were classified as having or not having MS according to the R-ATPIII [16], IDF [3] and JIS definitions. Under the R-ATPIII, a diagnosis of MS, requires three or more of the following criteria: 1) central obesity, defined as elevated waist circumference ≥102 cm for males or ≥88 cm for females; 2) elevated triglycerides ≥150 mg/dL; 3) reduced HDL-cholesterol <40 mg/dL in men or <50 mg/dL in women; 4) elevated blood pressure ≥130/85 mmHg or use of an antihypertensive agent; and 5) elevated fasting glucose ≥100 mg/dL or previous diagnosis of diabetes or impaired glucose tolerance. Diagnosis of MS according to the IDF definition requires the presence of abdominal obesity defined as elevated waist circumference ≥94 cm among men or ≥80 cm among women for European populations, together with at least two of the other four criteria of the R-ATPIII definition using the same categorical cut-off points. In much the same way, the JIS definition requires the presence of at least three of the five components and considers two levels of abdominal obesity for European populations, namely: a high cut-off point (102/88) for waist circumference proposed by European Cardiovascular Societies[23]; and a low cut-off point (94/80) as proposed by the IDF. As the JIS high cut-off point definition is identical to the R-ATPIII definition, only data for the JIS low cut-off point definition differ from those for the other former definitions of MS. Individuals on antihypertensive, diabetes and/or lipid-lowering medication were included in the elevated blood pressure, elevated fasting glucose and/or elevated triglyceride groups respectively.

Participants with fasting glucose ≥100 mg/dL and <126 mg/dL, not meeting the criteria for diabetes, were identified as having impaired fasting glucose [24]. Criteria for diabetes were the following: fasting glucose concentration ≥126 mg/dL; treatment for diabetes; or plasma HbA1c ≥7% [25].

### Statistical analysis

Prevalences and their 95% confidence intervals (CIs) were calculated for MS and each of its components. Prevalence estimates were standardised by age using the European standard population. A Wald test was used to test the significance of the differences observed between centrally obese and non-centrally obese subjects in terms of awareness of obesity as a health risk and associated lifestyles. For this purpose central obesity was defined with the high waist-circumference cut-off points (≥102 cm for men and ≥88 cm for women). All statistical analyses were performed using the STATA 10.0 software package (Stata Corporation, College Station, TX, USA).

## Results

Of the 2562 people invited to participate in the study, 61% agreed to give a fasting blood sample, yielding a final sample of 1555 (718 men and 837 women). The age and sex distribution of the sample was similar to that of the general population of the Murcia Region.

The main characteristics of the study population are shown in Table 1. While men displayed a worse profile than did women in terms of glucose status, lipids and smoking, women had a lower educational level and engaged in less physical activity.

**Table 1:** Characteristics of study subjects

	Men	Women	Total	p
	(n = 718)	(n = 837)	(n = 1555)	
**Age (years), mean ± SD**	47.2 ± 17.4	47.6 ± 18.0	47.4 ± 17.7	0.622
20-29%	18.8	18.8	18.8	0.799
30-39	21.6	22.5	22.1	
40-49	17.8	16.5	17.1	
50-59	13.7	13.9	13.8	
60-69	14.2	12.5	13.3	
70+	13.9	15.9	15.0	
				
**Residence, %**				
Urban	74.0	76.9	75.6	0.172
Rural	26.0	23.1	24.4	
				
**Education, %**				
Little or no formal education	21.1	29.1	25.4	0.004
Primary	20.3	17.0	18.5	
Secondary	39.1	35.6	37.2	
University	19.6	18.3	18.9	
				
**Waist circumference (cm), mean ± SD**	97.5 ± 10.8	87.3 ± 13.4	92.1 ± 13.3	<0.001
				
**Systolic BP (mmHg), mean ± SD**	134.4 ± 20.6	122.5 ± 24.0	128.0 ± 23.3	<0.001
**Diastolic BP (mmHg), mean ± SD**	80.4 ± 11.2	74.2 ± 10.3	77.0 ± 11.2	<0.001
				
**Fasting glucose (mg/dl), mean ± SD**	99.5 ± 29.8	93.4 ± 25.3	96.2 ± 27.6	<0.001
**HDL-Cholesterol (mg/dl), mean ± SD**	49.3 ± 11.2	59.6 ± 13.5	54.9 ± 13.5	<0.001
**Triglycerides (mg/dl), mean ± SD**	128.9 ± 111.2	92.5 ± 51.4	109.3 ± 86.4	<0.001
				
**Glucose status, %**				
Normoglycaemia	68.0	78.7	73.8	<0.001
Impaired fasting glucose	19.5	11.7	15.3	
Diabetes	12.5	9.6	10.9	
				
**Smoking, %**				
Non-smoker	46.3	65.4	56.6	<0.001
Ex-smoker	15.0	4.1	9.1	
Current smoker	38.7	30.5	34.3	
				
**Leisure-time physical activity**				
Household chores (hours/week), mean ± SD	3.8 ± 6.4	22.9 ± 17.3	14.1 ± 16.5	<0.001
Walking (hours/week), mean ± SD	6.9 ± 7.3	5.9 ± 6.1	6.4 ± 6.7	0.003
Moderate/vigorous PA (hours/week), mean ± SD	1.9 ± 3.8	0.9 ± 2.2	1.4 ± 3.1	<0.001

SD: standard deviation. BP: Blood pressure. HDL: High-density lipoprotein. PA: Physical activity

Table 2 shows the prevalence of MS and each of its components across categories of sex, age, residence, education and glucose status. Prevalence of MS varied from 27.2% (95%CI: 25.2-29.2) to 32.2% (95%CI: 30.1-34.3) and 33.2% (95%CI: 31.2-35.3), according to the R-ATPIII, IDF and JIS94/80 respectively. Whereas the prevalence of MS according to the IDF and JIS94/80 definitions increased with age to a figure of approximately 60% in persons aged 70 years and over, prevalence of R-ATPIII MS seemed to increase until age 60 years and then stabilise at around 53% in persons aged 60 years and over. The most common cardiovascular risk factors and components of MS in our population were hypertension (46.6%) and central obesity (40.7% or 66.1%, according to high or low waist circumference cut-off points respectively). Prevalence of MS and each of its components increased markedly with age (*p *for trend <0.001). Prevalence of MS based on any of the three definitions, and prevalence of each of its components were both higher in persons with little or no formal education (51.7% R-ATPIII, 56.4% IDF and 57.3% JIS94/80) and glucose disorders.

**Table 2:** Prevalence (%) of metabolic syndrome and its components according to the R-ATPIII, IDF and JIS94/80 criteria

	Elevated waist circumference 102/88^1^	Elevated waist circumference 94/80^2^	Elevated triglycerides	Reduced HDL-cholesterol	Elevated blood pressure	Elevated fasting glucose	Metabolic syndrome R-ATPIII	Metabolic syndrome IDF	Metabolic syndrome JIS94/80
	%	%	%	%	%	%	%	%	%
	(95%CI)	(95%CI)	(95%CI)	(95%CI)	(95%CI)	(95%CI)	(95%CI)	(95%CI)	(95%CI)
**Total unadjusted**	40.7	66.1	22.8	27.3	46.6	26.0	27.2	32.2	33.2
	(38.6-42.7)	(64.1-68.2)	(20.8-24.7)	(25.1-29.4)	(44.6-48.6)	(24.1-28.0)	(25.2-29.2)	(30.1-34.3)	(31.2-35.3)
**Total age-adjusted**	39.9	65.8	22.7	27.0	44.9	25.0	26.1	31.1	32.1
**European population**	(37.8-42.0)	(63.7-67.9)	(20.7-24.7)	(24.8-29.2)	(42.8-47.0)	(23.1-27.0)	(24.1-28.2)	(28.9-33.2)	(29.9-34.2)
									
**Sex**									
Men	33.8	63.7	29.0	23.7	57.7	32.0	28.2	36.9	38.9
	(30.6-37.0)	(60.5-66.9)	(25.7-32.2)	(20.6-26.8)	(54.4-61.0)	(29.0-35.1)	(25.1-31.2)	(33.6-40.2)	(35.6-42.2)
Women	46.6	68.3	17.4	30.3	37.1	20.9	26.3	28.1	28.4
	(44.0-49.2)	(65.7-70.9)	(15.1-19.8)	(27.3-33.4)	(34.8-39.5)	(18.5-23.3)	(23.8-28.9)	(25.5-30.8)	(25.8-31.0)
**Age, years**									
20-29	10.7	27.8	8.2	16.8	16.5	3.8	3.4	6.2	6.2
	(7.1-14.2)	(22.8-32.9)	(5.1-11.3)	(12.6-21.0)	(12.3-20.6)	(1.6-5.9)	(1.3-5.5)	(3.4-9.0)	(3.4-9.0)
30-39	22.3	53.7	20.7	25.4	23.1	8.7	12.5	18.1	19.2
	(17.9-26.7)	(48.4-58.9)	(16.8-24.6)	(20.8-30.0)	(19.0-27.2)	(5.8-11.7)	(9.1-15.9)	(14.2-22.1)	(15.2-23.3)
40-49	33.8	69.6	22.2	26.3	38.3	22.9	21.1	29.5	30.2
	(28.1-39.5)	(63.9-75.2)	(17.4-27.0)	(21.1-31.5)	(33.0-43.5)	(18.2-27.7)	(16.3-26.0)	(24.3-34.8)	(24.9-35.5)
50-59	59.2	85.0	31.3	30.4	58.0	35.5	37.4	39.6	42.1
	(53.4-64.9)	(80.4-89.6)	(25.0-37.6)	(24.1-36.7)	(51.5-64.6)	(29.2-41.9)	(30.8-43.9)	(33.0-46.3)	(35.4-48.7)
60-69	71.4	92.2	32.4	37.7	80.2	49.8	53.6	57.8	58.9
	(65.4-77.3)	(88.7-95.7)	(26.1-38.6)	(31.4-44.0)	(74.9-85.5)	(42.9-56.6)	(46.9-60.3)	(51.0-64.5)	(52.3-65.6)
70+	69.3	88.6	28.3	32.2	88.7	53.2	52.6	59.6	60.3
	(64.2-74.4)	(84.6-92.6)	(22.7-34.0)	(26.4-38.0)	(84.6-92.8)	(47.0-59.5)	(46.4-58.7)	(53.5-65.8)	(54.2-66.5)
**Residence**									
Urban	39.7	64.9	21.7	26.9	45.0	25.3	26.1	30.7	31.7
	(37.4-42.1)	(62.5-67.3)	(19.5-23.9)	(24.4-29.4)	(42.8-47.3)	(23.1-27.5)	(23.9-28.4)	(28.4-33.1)	(29.4-34.1)
Rural	43.5	69.9	26.1	28.4	51.6	28.4	30.5	36.8	37.9
	(39.5-47.6)	(66.0-73.8)	(21.9-30.2)	(24.1-32.7)	(47.5-55.7)	(24.3-32.5)	(26.5-34.6)	(32.4-41.1)	(33.5-42.3)
**Education**									
Little or no formal education	72.8	90.7	33.0	37.3	79.1	45.4	51.7	56.4	57.3
	(68.7-76.8)	(88.0-93.5)	(28.4-37.5)	(32.7-41.9)	(75.2-83.0)	(40.6-50.3)	(46.8-56.5)	(51.6-61.3)	(52.4-62.1)
Primary	49.8	79.3	23.7	26.5	55.8	33.8	34.1	38.2	39.4
	(44.1-55.5)	(74.6-84.0)	(18.8-28.6)	(21.4-31.5)	(50.2-61.4)	(28.5-39.1)	(28.7-39.6)	(32.7-43.8)	(33.8-44.9)
Secondary	25.2	53.4	18.4	25.1	29.4	13.7	14.4	20.0	21.2
	(21.7-28.7)	(49.4-57.4)	(15.3-21.4)	(21.6-28.7)	(25.8-33.0)	(11.0-16.4)	(11.6-17.2)	(16.8-23.2)	(17.9-24.4)
University	19.6	45.7	16.7	18.4	28.5	16.7	12.7	18.6	18.8
	(15.1-24.1)	(40.2-51.2)	(12.6-20.9)	(14.0-22.9)	(23.5-33.5)	(12.6-20.8)	(8.9-16.4)	(14.2-22.9)	(14.5-23.2)
**Glucose status**									
Normoglycaemia	31.8	58.2	16.9	24.0	35.6	0.0	11.8	15.9	16.1
	(29.4-34.2)	(55.6-60.8)	(14.8-19.0)	(21.5-26.4)	(33.1-38.1)		(10.0-13.6)	(13.9-18.0)	(14.1-18.2)
Impaired fasting glucose	61.3	87.7	34.5	31.5	73.3	100	66.2	76.5	78.9
	(55.2-67.3)	(83.5-91.9)	(28.5-40.4)	(25.6-37.5)	(67.7-78.9)		(60.3-72.2)	(71.1-81.9)	(73.8-84.0)
Diabetes	72.3	90.4	45.9	43.5	84.0	98.2	76.9	81.9	85.2
	(65.5-79.1)	(85.9-94.8)	38.4-53.3)	(36.2-50.9)	(78.5-89.5)	(96.3-100)	(70.7-83.2)	(76.1-87.8)	(79.9-90.5)

R-ATPIII: Revised National Cholesterol Education Program Adult Treatment Panel III. IDF: International Diabetes Federation.

JIS 94/80: Joint Interim Statement with waist-circumference cut-off points of ≥94 cm for men and ≥80 cm for women

^1^Waist circumference ≥102 cm for men and ≥88 cm for women

^2^Waist circumference ≥94 cm for men and ≥80 cm for women

**Figure 1:**
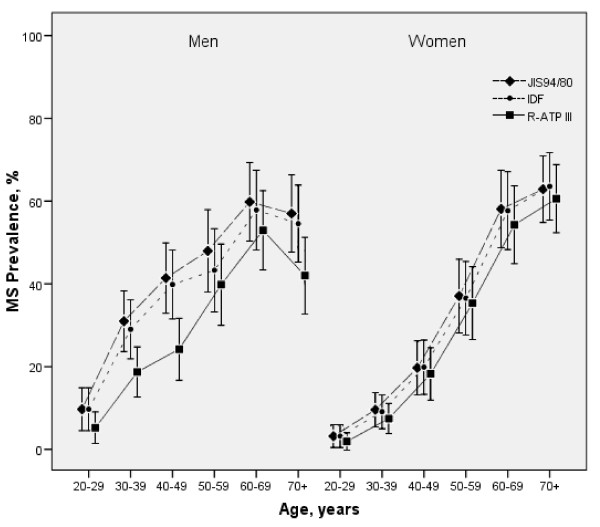
**Prevalence and 95%CI of metabolic syndrome according to the R-ATPIII, IDF and JIS94/80 definitions, by sex and age groups**. R-ATPIII: Revised National Cholesterol Education Program Adult Treatment Panel III. JIS 94/80: Joint Interim Statement with waist-circumference cut-off points of ≥94 cm for men and ≥80 cm for women. IDF: International Diabetes Federation.

As can be seen from Figure 1, regardless of the definition used, prevalence of MS increased with age, except for men aged 70 years and over, who registered a lower estimated prevalence than did their counterparts in the 60-69 age group. As the IDF and JIS94/80 definitions use lower waist-circumference cut-offs, the estimates of MS prevalence with these definitions were higher in each age and sex group than were the corresponding R-ATPIII/JIS102/88 estimates, with these differences being greater among men.

**Figure 2:**
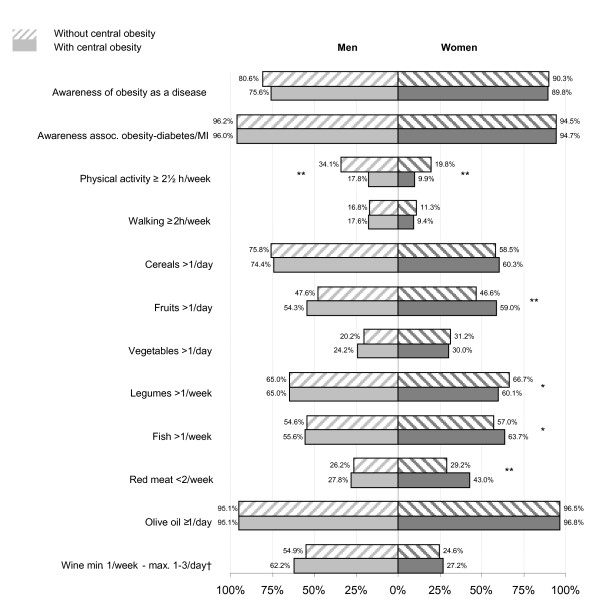
**Awareness of the pathogenic role of obesity and adherence to preventive recommendations for adequate weight control**. Central obesity was defined with the high waist-circumference cut-off points (≥102 cm for men and ≥88 cm for women). ^a^Wine intake from a minimum of 1/week until a maximum of 3/day in men and 1/day in women. * p < 0.05. ** p < 0.01.

Figure 2 depicts the degree of awareness of the pathogenic role of obesity and adherence to preventive recommendations and adequate weight control. Although most persons were aware that obesity increased health risks, regular exercise was very unusual (13.0% centrally obese, 27.2% non-centrally obese). The proportion of women doing the recommended two and a half hours of moderate/vigorous physical exercise weekly was extremely low, both for centrally obese (9.9%) and non-centrally obese subjects (19.8%), and amounted to half the corresponding proportions among the men. Only 17% of men and 10% of women reported walking at least two hours per week, with practically no difference between centrally obese and non-centrally obese subjects. Frequency of eating the main dietary components was similar among centrally obese and non-centrally obese persons. Only 21% of men and 31% of women reported eating vegetables more than once daily, and 27% of men and 35% of women consumed red meat less than twice per week. The proportion of women following the recommended frequency of eating fruit, fish and red meat proved slightly higher among centrally obese than non-centrally obese persons. At the opposite extreme, about 95% of persons used olive oil daily, irrespective of sex or obese status.

## Discussion

In the Murcia Region, one-third of the general population aged 20 years and over met the IDF and JIS94/80 definitions for MS, which was slightly higher than the proportion yielded by the R-ATPIII definition. The crude and European population age-adjusted MS prevalence figures were very similar, namely, 27.2% vs. 26.1%, 32.2% vs. 31.1% and 33.2% vs. 32.1% according to the R-ATPIII, IDF and JIS94/80 definitions respectively.

Our findings on the prevalence of MS are comparable to those reported both by Qiao's study [26] based on collaboratively analysed data from nine European cohort studies, and by other studies undertaken in Northern European countries [27,28]. The prevalence in our study was higher than that encountered by other studies conducted in Australia [29], Russia [30], Italy [31] or even other Spanish regions [32-34] in which it ranged from 15 to 22%. Even so, the prevalence estimated by us was lower than that for the US population [35]. To a certain extent, such differences in MS prevalence among studies are due to differences in methodology and, more particularly, in the measures of obesity used. A recent study by Vega et al. [36] showed that prevalence of MS was influenced by the measure of obesity used, and went on to question the inclusion of any measure of obesity among the diagnostic components of MS. The so-called abridged ATPIII MS - defined as 3 of 4 independent risk factors after obesity is removed from the diagnostic criteria - successfully identified a subgroup of patients who were at higher risk of cardiovascular disease and were therefore candidates for intensive cardiovascular risk reduction. Moreover, regardless of the definition used, MS identifies a group of subjects at significantly greater coronary heart disease risk, and the proportion of such higher-risk subjects is comparable regardless of whether the high or low waist-circumference cut-off point is used, as was recently shown by Alkerwi et al. [37]. In contrast, overweight and obesity measured by reference to body mass index failed to detect any increased cardiovascular risk in our population [6].

In agreement with Martínez-Candela's study [34], conducted on a similar population, and with the studies undertaken by Halldin in Sweden [27], and Alkerwi in Luxembourg [37], prevalence of MS was higher in men than in women. This finding differs from other studies which reported similar prevalences in both sexes [28,35,38] or, on the contrary, a higher prevalence in women then in men [30,31,33,39]. As with other studies, prevalence of MS and of each of its components was observed to increase with age and be higher among the population with a low education level. The results of our study are in line with several other studies [29,40] that have reported a levelling-off or even a decline in the prevalence of MS among men after the sixth or seventh decade of life versus a linear increase with age among women. This difference may be due to selective survival with increased mortality rates among men in general, and among those with metabolic risk factors in particular.

Although most persons were aware of the pathogenic role of obesity, only a small proportion reported physical activity and dietary habits consistent with the preventive recommendations for weight control. While the lowest adherence to the dietary recommendations was found for vegetables and red meat, the highest adherence was found for olive oil. The food consumption patterns reported by centrally obese and non-centrally obese persons were very similar, except for the higher frequency of fruit and fish intake and lower frequency of red meat intake among centrally obese women.

Substantial differences in physical activity between the centrally obese and non-centrally obese were observed for men and women alike, with the proportion of non-centrally obese persons doing the recommended physical activity being twice as high as the corresponding proportion of centrally obese persons. This is in agreement with Halldin et al's study [27], which observed a strong inverse dose-response association between leisure-time physical activity and MS, and stressed the role of physical activity in the prevention and treatment of MS. Even in the non-centrally obese population, the proportion of persons with an adequate frequency of physical activity is excessively low, especially among women. A possible explanation for the extremely low proportion of women doing the recommended physical activity could be the long hours that women devote to household chores (an average of 23 hours per week for women vs. 4 hours per week for men), which thus competes with the time available for physical activity.

This study has several limitations. First, the 61% blood-extraction response rate was less than desirable. Nevertheless, as the sex- and age-distribution of the sample was similar to that of the general population, selection bias can be assumed to be minimal. Secondly, while the use of a food-frequency questionnaire without any quantitative or semi-quantitative measure hinders the assessment of energy intake, an important factor in the appearance and maintenance of obesity, it nevertheless allows for comparison with the Mediterranean diet pyramid [20]. Thirdly, due to the study's cross-sectional design, the dietary pattern observed in the centrally obese population may, to a certain extent, reflect changes in diet introduced as a result of medical advice. Finally, a misreporting bias cannot be ruled out, since obese subjects are known to be prone to overestimate their adherence to healthy lifestyles, e.g., by underreporting their energy intake and overreporting their intake of fruit and vegetables [41].

The strengths of our study include the fact that it benefits from being a population-based study covering a wide age range, and that prevalence of MS was estimated in accordance with the latest, most widely used definitions. Moreover, by examining both degree of awareness of the pathogenic role of obesity and adherence to preventive recommendations and adequate weight control, we were able to identify the discrepancy between the high degree of awareness and its low impact on improving adherence to healthier lifestyles. The study was based on the most recent data on cardiovascular risk factors available in our region and provides valuable information for decision-makers with regard to areas of health promotion that should be reinforced. Measures targeted at improving adherence to preventive recommendations and adequate weight promotion are required in both sexes, with special stress on dietary recommendations in men and increased physical activity in women.

Further studies are called for to assess trends in cardiovascular risk factor prevalence and changes in lifestyles in our region, and their impact on risk of coronary heart disease. Using a low cut-off point for waist circumference identifies more persons who need intervention; and, as this might prove excessive, further studies are needed to ascertain which waist-circumference cut-off point would be more appropriate for our population. Similarly, longitudinal studies are warranted to assess the impact of MS and abdominal obesity on cardiovascular risk among the local population.

## Conclusions

The prevalence of MS is high in our population, is comparable to that found in northern Europe, and varies with the definition used. Adherence to preventive recommendations and adequate weight promotion is very low. In the absence of a specific treatment for MS, integrated intervention based on a sustained increase of physical activity and changes in diet should be reinforced.

## Competing interests

The authors declare that they have no competing interests.

## Authors' contributions

DG performed the statistical analyses and drafted the manuscript. DS and JMH conducted a critical review of the manuscript and helped with the statistical analyses. JMEC and APM performed the clinical analysis and contributed to data interpretation and the critical review of the manuscript. CN was involved in the study design, critically reviewing and revising the manuscript, made substantial contributions to its final content, and obtained funding. MJT was involved in study conception and design, co-ordinated the field work, participated in the analysis and in the drafting of the manuscript, and obtained funding. All authors read and approved the final manuscript.

## Pre-publication history

The pre-publication history for this paper can be accessed here:

http://www.biomedcentral.com/1471-2458/11/562/prepub
